# Guillain-Barre Syndrome Presenting With Bilateral Facial Nerve Palsy

**Published:** 2014

**Authors:** Sorour INALOO, Pegah KATIBEH

**Affiliations:** 1 Pediatric Department, Shiraz University of Medical Sciences, Shiraz, Iran

**Keywords:** Guillain-Barre syndrome, Facial diplegia, Bilateral facial palsy, Bell’s palsy

## Abstract

**Objective:**

This case study is about an 11-year-old girl with bilateral facial weakness, abnormal taste sensation, and absent deep tendon reflexes of both knees and ankles. However, the muscle power of the lower and upper extremities across all muscle groups was normal. After 2 days, she developed paresthesia and numbness in the lower extremities. Other neurologic examinations, such as fundoscopic evaluation of the retina were normal with the muscle power of both upper- and lower-extremities intact. A lumbar puncture revealed albumincytological dissociation. EMG and NCV were in favor of Guillain-Barre syndrome, for which IVIG was prescribed and the abnormal sensations in the lower limbs rapidly improved. Bilateral facial diplegia without weakness and paresthesia is a variant of Guillain-Barre syndrome that mostly presents with acute onset, rapid progression with or without limb weakness, paresthesia, and decreased or absent DTR and albumin-cytological dissociation.

## Introduction

Guillain-Barré syndrome (GBS) is an acute inflammatory demyelinating peripheral neuronal disorder. Facial nerve involvement has been reported in about 50% of GBS patients. This is unlike unilateral facial nerve palsy and bilateral involvement is considered an unusual presentation. Patients, who present with bilateral facial nerve palsy, are mostly investigated and sometimes are managed for etiologies other than GBS, usually as idiopathic (Bell’s) palsy. However, differential diagnosis such as GBS, diabetes mellitus, bacterial meningitis, infectious mononucleosis, sarcoidosis, human immunodeficiency virus (HIV) infection, Lyme disease, syphilis, and leprosy should be kept in mind. Different outcomes and the management of these various diagnoses warrants stepwise workups to exclude probable etiologies such as GBS(1-2).

## Case Report

The presenting case is an 11-year-old girl with bilateral facial weakness from oneday prior to her referral to our center. She had a history of upper respiratory tract infection that was treated with a routine therapeutic dose of acetaminophen and coamoxiclave.

There were no other significant issues in her history except an earache two-days prior to her facial weakness. The patient had no finding or history of trauma or tick exposure. In a physical examination mask face, a complete bilateral facial weakness involving the forehead (grade 5 according to House-Brackmann score) and abnormal taste sensation were obvious. Other cranial nerves as well as motor and sensory nervous systems were intact. However, deep tendon reflexes of both knees and ankles were absent. The muscle power of the lower and upper extremities across all the muscle groups was normal. No bowel or bladder dysfunction was reported and other neurologic examinations including fundoscopic evaluation of the retina along with examination of the ears and tympanometry were shown to be normal. Routine laboratory examinations, blood culture, throat culture were unremarkable and serum assays for anti-neutrophil antibody (ANA), anti-doublestrand DNA antibody (ds DNA), anti-phospholipid antibody (ACLA), anti neurophil cytoplasmic antibody (ANCA), CRP, and ESR were in normal ranges.

Polymerase chain reaction (PCR) examination of the blood and saliva for herpes and cytomegalovirus (CMV) viruses, and viral capsid antigen for EBV were all negative. The brain MRI and chest x-ray were normal. 

Electromyography (EMG) and nerve conduction velocity (NCV) revealed a demyelinating type of facial palsy. EMG and NCV of extremities were also in favor of demyelinating motor polyneuropathy suggestive of GBS. Eye protection with artificial tears during the day and the use of jelly and eye patches at night was advised.

However, after two days she developed paresthesia and numbness in the lower extremities. Other neurologic examinations, fundoscopy and muscle power of both the upper and lower extremities were still intact. A lumbar puncture was done and the result of cerebrospinal fluid (CSF) analysis was reported as below: CSF glucose: 84 mg/dl, (CSF) protein: 123 mg/dl but without any cell, typical protein-cell count pattern (albumin–cytological dissociation) for GBS. CSF culture and CSF examination with PCR for herpes were negative. EMG and NCV were requested again, which was suggestive of GBS due to delayed recorded waves and a demyelinating motor polyneuropathy. According to these results, intravenous immunoglobulin (IVIG) 2 g/kg for a 5-day period was started for the patient. Fortunately, the abnormal sensation in her lower limb rapidly improved, but she returned home with residual bilateral facial palsy. After 3 weeks of follow up, the right side of her face showed relative improvement without rehabilitation and 2 months later, she had normal DTR of both lower limbs. 

The residual bilateral facial palsy (grade 4 according to House-Brackmann score) was recovered completely after eight months.

## Discussion

Bilateral facial palsy is not considered a common clinical presentation. However, it might appear secondary to systemic diseases such as Lyme, GBS, sarcoidosis, HIV infection, leukemia, Möbius syndrome, Kawasaki, mycoplasma infection, diabetes, borreliois, infection with herpes virus, fracture in base of skull, syphilis, pontine glioma, pregnancy, leprosy, mononucleosis, linzolid therapy, systemic lupus erythematosis, cryptococcal meningitis, pontine tegmental hemorrhage, and bulbospinal muscular atrophy (3-7). 

Among the above-mentioned etiologies, GBS is a life threatening diseases that requires urgent medical management. It usually presents with bilateral symmetrical ascending flaccid paralysis but other unusual presentations such as cranial nerve palsy have been reported and among these, facial palsy is the most common (24-60%). Bilateral simultaneous facial palsy is increasingly recognized as an atypical variant of GBS in adults (8,-10). Facial diplegia distal limb paresthesias, sixth nerve palsy, bilateral lumbar polyradiculopathy, and a combination of Fisher’s syndrome and pharyngealcervical-brachial weakness along with facial diplegia with hyporeflexia are considered GBS variants (11). 

Third nerve palsy can also be a rare presentation of GBS (12). Rarely GBS has been reported to present as unilateral facial palsy (13).

From 20 to 60% of patients with GBS develop facial palsy that is usually bilateral but mostly associated with limb weakness. Landry Guillain-Barre Syndrome represents a probably infectious polyneuritis that involves primary peripheral nerves that are bulbar, myelitic, and cerebral variants. Bilateral facial diplegia without weakness and paresthesia is a new but rare variant of GBS that typically presents with rapid progressive acute onset paresthesia, decreased or absent DTR and albumin-cytological dissociation with, or without extremity weakness (11).

Isolated facial diplegia indicates poor prognosis (14). 

The aforementioned case presented with bilateral facial palsy, absent DTR, normal muscle power, and increased CSF protein without pleocytosis. Nearly all other causes of bilateral facial palsy were ruled out by specific investigation along with a comparison with other findings in physical examinations. Lyme is the most common cause of bilateral facial nerve palsy (6), but it is not endemic to Iran or complied with the clinical presentation of the patient. 

Bilateral facial palsy should be considered in children as well as adults as a variant of GBS based on other clinical clues. Appropriate treatment for this variant is yet to be determined but it is usually treated as GBS.
